# Forest fragmentation modifies the composition of bumblebee communities and modulates their trophic and competitive interactions for pollination

**DOI:** 10.1038/s41598-020-67447-y

**Published:** 2020-07-02

**Authors:** Carmelo Gómez-Martínez, Anne Lene T. O. Aase, Ørjan Totland, Javier Rodríguez-Pérez, Tone Birkemoe, Anne Sverdrup-Thygeson, Amparo Lázaro

**Affiliations:** 1 Mediterranean Institute for Advanced Studies (UIB-CSIC), Global Change Research Group, C/Miquel Marquès 21, 07190 Esporles, Balearic Islands Spain; 2 Faculty of Environmental Sciences and Natural Resource Management, Norwegian University of Life Sciences, P.O. Box 5003, 1432 Ås, Norway; 3 Department of Biological Sciences, University of Bergen, P.O. Box 7800, 5020 Bergen, Norway; 4 IMAB (Institute for Multidisciplinary Research in Applied Biology), Departamento Ciencias del Medio Natural, Centro Jerónimo de Ayanz, Universidad Pública de Navarra (UPNA), Campus Arrosadía, 31006 Pamplona, Navarra Spain

**Keywords:** Plant sciences, Systems biology, Zoology, Animal behaviour, Entomology, Ecology, Agroecology, Behavioural ecology, Biodiversity, Community ecology, Conservation biology, Ecological networks, Ecosystem services, Forest ecology, Grassland ecology

## Abstract

Understanding the effects of landscape fragmentation on global bumblebee declines requires going beyond estimates of abundance and richness and evaluating changes in community composition and trophic and competitive interactions. We studied the effects of forest fragmentation in a Scandinavian landscape that combines temperate forests and croplands. For that, we evaluated how forest fragmentation features (patch size, isolation and shape complexity, percentage of forest in the surroundings) as well as local flowering communities influenced bumblebee abundance, richness and community composition in 24 forest patches along a fragmentation gradient. In addition, we assessed the effect of fragmentation on bumblebee–plant network specialization (*H*_2_*′*), and potential inter- and intraspecific competition via shared plants. Patch isolation was associated with lower bumblebee abundance, whereas flower density was positively related to both bumblebee abundance and richness. Overall, forest fragmentation reduced the abundance of forest-specialists while increasing the abundance of open-habitat species. Patches with complex shapes and few flowers showed more generalized bumblebee–plant networks (i.e., fewer specific interactions). Patch shape complexity and the percentage of forest also modified inter- and intraspecific competitive interactions, with habitat generalists outcompeting forest specialists in fragmented areas. Understanding these mechanisms is necessary to anticipate to the impact of forest fragmentation on bumblebee decline.

## Introduction

**Figure 1:**
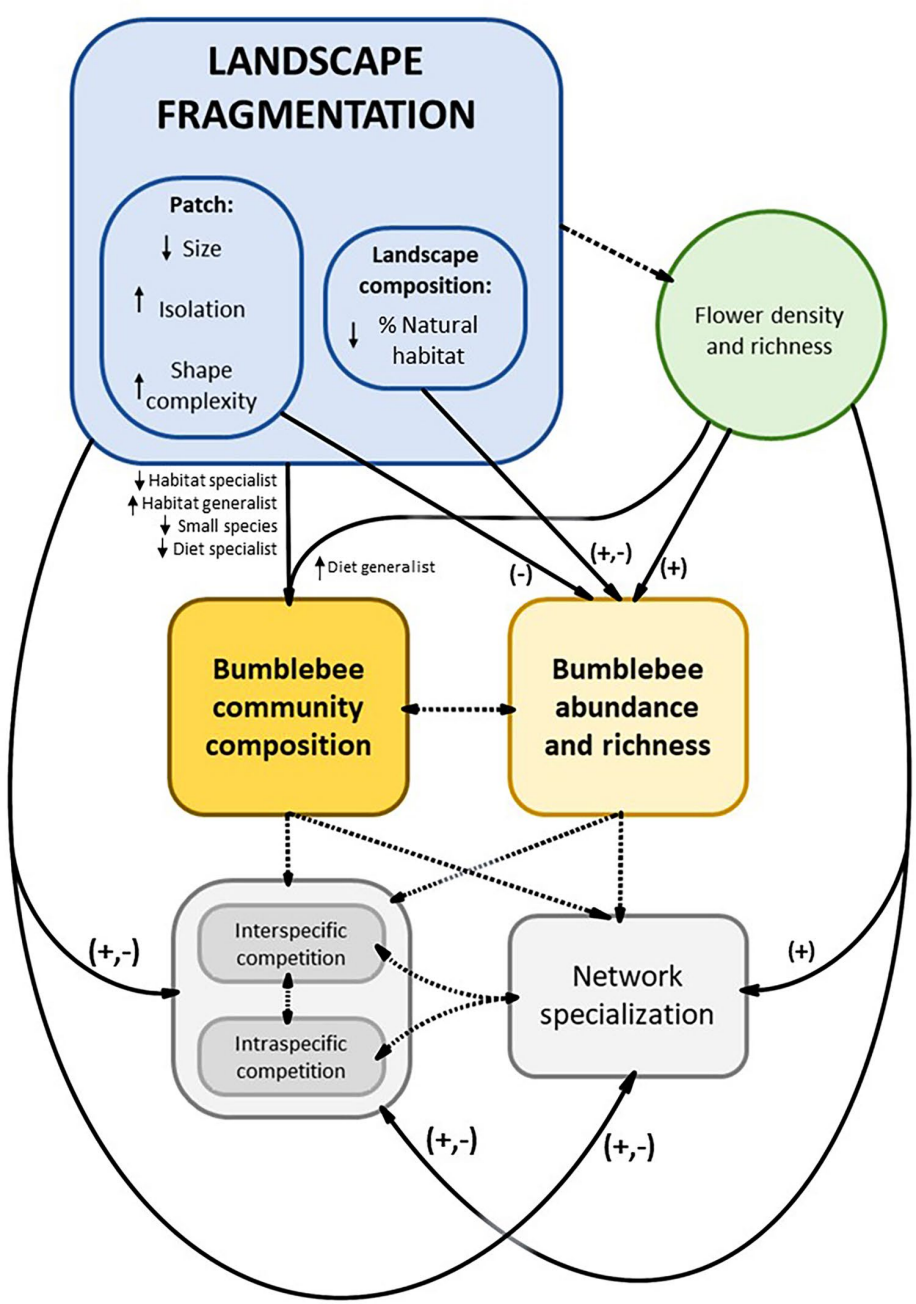
Conceptual diagram. Summary of the expected relationships between landscape fragmentation and the composition of bumblebee communities and their trophic and competitive interactions. Solid lines correspond to relationships addressed in this study, and symbols represent the expected direction of these relationships according to the literature (+: positive; −: negative). Dashed lines correspond to potential relationships not addressed in this study.

Bumblebees are essential for crop and wildflower pollination in temperate latitudes, where many plant species are pollinated primarily by them^1^. However, these important pollinators are suffering worldwide declines^1,2^, which have been mainly attributed to the loss and fragmentation of natural and semi-natural areas due to land-use changes^1,3^. Such negative effects of habitat fragmentation are related to the reduction in the extent of natural habitats^1^, to changes in the quantity and quality of flowering resources^4,5^ and nesting sites^6^ and their spatial configuration^7^, as well as to changes in habitat edge/area relationships that could affect habitat suitability^8^ (see Fig. 1 for a conceptual diagram).

Despite the overall bumblebee declines, not all the species may respond similarly to landscape fragmentation. Indeed, previous studies have shown that while some species have considerably declined in fragmented landscapes, others have remained relatively abundant^9,10^. The uneven effects of landscape fragmentation on bumblebee species may depend, among other aspects, on their habitat preferences, foraging ranges, and behavioural/morphological feeding adaptations^3,11^ (Fig. 1). For instance, forest specialist bumblebee species or those that nest above-ground may be negatively affected by forest fragmentation, while other species adapted to open areas that nest below ground might benefit^11,12^. Smaller species may be also more affected by the isolation of suitable habitat patches^13^, because specific foraging ranges^14^ are known to increase with body size^15,16^. Regarding diet specialization, bumblebee species with narrower niche breath, such as long-tongued bumblebees that feed preferentially on flowers with deep corolla tubes, may be more vulnerable to habitat reduction than short-tongued ones with wider diet preferences^9,10^. These species-specific responses of bumblebees to landscape fragmentation may result in different bumblebee communities along fragmentation gradients (Fig. 1). However, the patterns of variation in the composition of bumblebee communities along these gradients are still little explored.

Furthermore, landscape fragmentation may influence bumblebee–plant trophic interactions and affect the structure of pollination networks because bumblebee feeding choices vary with the availability of flowering resources^17,18^ and competitors^19–21^ (Fig. 1). However, literature on the response of plant–pollinator networks to habitat loss and disturbance reports inconclusive results. Some of these studies showed that habitat loss and disturbance lead to a decrease in plant–pollinator interactions and pollinator diversity^22–26^, and to an increase in network generalization^23,26^, which might be driven by a loss of specialist species^27^ and/or the loss of specialized interactions^28,29^. However, some other studies showed a lack of relationship between habitat disturbance and network structure^30,31^, or reported an increase in pollinator specialization with habitat loss^25,32,33^ attributed to niche partitioning, as the pollinators might narrow their diet to avoid competition if competitor abundance is high^32^. Specialization though niche partitioning might also increase with the amount of flowering resources, as often occurs with the appearance of new resources^34^ (Fig. 1).

However, not only the specialization of bumblebee–plant interactions might change along fragmentation gradients. The way bumblebees share the plant species they pollinate might also vary, influencing the potential for intra- and interspecific competition (Fig. 1). As a general rule, it could be expected that the most abundant bumblebee species in a community might have a higher potential to influence other bumblebees through shared plants^35,36^, especially if they are generalist species or when they share traits to efficiently exploit the same type of flowering resources^37^. In addition, a negative relationship between inter- and intraspecific competition could be expected (Fig. 1), as the effect of competitors on realized niche breadth occurs both at the interspecific^19,38^ and at the intraspecific level^39^. These competitive interactions might be further modulated by changes in the flowering resources. For instance, in a flower-impoverished landscape, the limited possibilities for competition avoidance^38^ might increase the strength of the competition^40^ (Fig. 1).

The aim of this study was to investigate how bumblebee communities and their trophic and competitive interactions responded to forest fragmentation in an agricultural landscape of southern Norway. For that, we recorded bumblebee visits to flowers, as well as flower richness and density, during a whole summer, in 24 forest patches, differing in size, isolation, patch shape complexity and the percentage of forest that surrounded them (SI Table S1). Previous work in this system showed that patch shape complexity was related to a higher density and diversity of flowers, while flower density decreased with the percentage of forest in the surroundings^5^. We also accounted for phenological variations in bumblebee abundance and richness which are often strong in temperate systems^41,42^ and may influence their response to changes in local flower communities^41^ and landscape characteristics^42^. Specifically, we assessed whether forest fragmentation: (1) reduced overall bumblebee abundance (number of visits) and richness (number of species); (2) influenced community composition, by reducing the abundance of forest-specialist species and increasing the abundance of habitat-generalist ones; (3) reduced specialization of bumblebee–plant networks (*H*_2_*′*); and (4) modified bumblebees’ inter- and intraspecific competition for pollination, as a result of changes in the relative abundances of both bumblebees and resources.

## Results

We registered a total of 861 bumblebee visits during 372 sampling days. Most of them (ca. 98%) belonged to one of the following ten species: *Bombus pascuorum* (279 records), *B. lucorum* /*terrestris* (255), *B. wurflenii* (74), *B. lapidarius* (67), *B. hortorum* (56), *B. hypnorum* (54)*, B. pratorum* (47)*, B. sylvarum* (11), *B. jonellus* (9)*,* and *B. soroeensis* (1). SI Tables S2 and S3 show detailed information on the recorded number of visits and species per study forest patch, as well as the standardized estimates of bumblebee abundance and richness used in the analyses.

### Bumblebee abundance and richness

**Figure 2:**
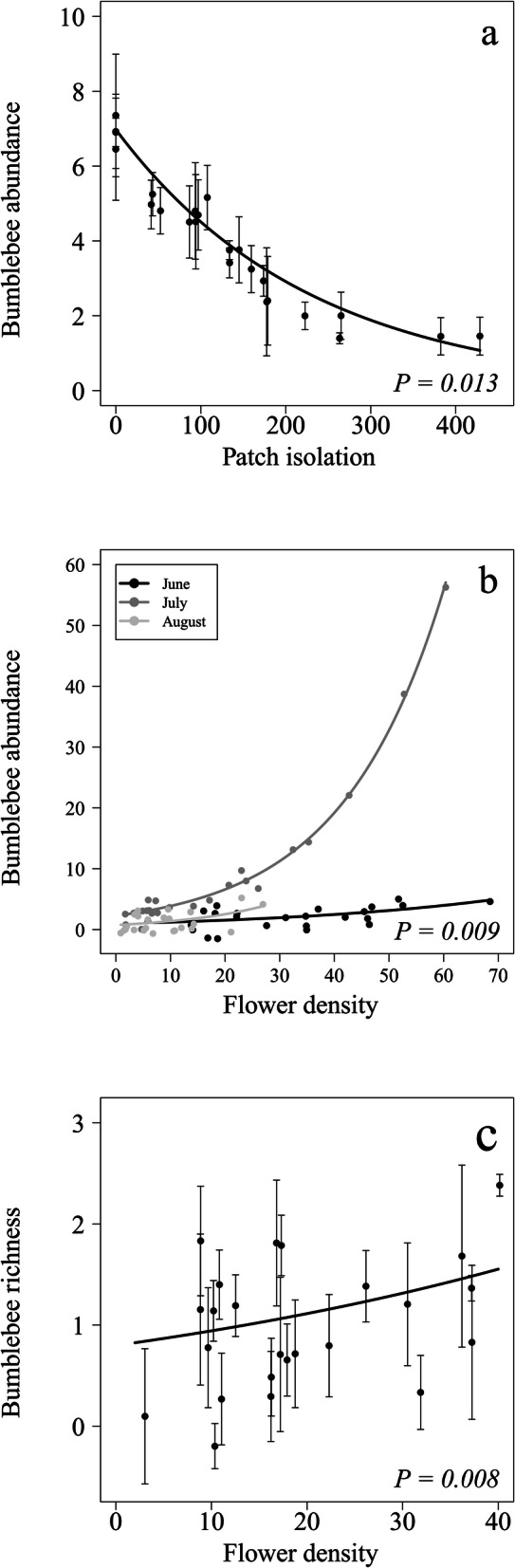
Forest fragmentation and bumblebee abundance and richness. Partial residual plots showing the relationships between (**a**) bumblebee abundance and patch isolation; (**b**) bumblebee abundance and flower density; and (**c**) bumblebee richness and flower density. Lines represent the estimates of the best models, the dots represent average partial residuals for each study forest patch, and vertical lines the standard errors. Whenever an interaction was significant, the estimates were plotted separately for each sampling month.

The best model showed that bumblebee abundance decreased with patch isolation (Table 1a; Fig. 2a) and increased with flower density, although the strength of this relationship depended on the month, being steeper in July than in June or August (Table 1a; Fig. 2b).

**Table 1:** Results of the best models showing the relationships between landscape characteristics and the local flowering community and (a) bumblebee abundance, (b) bumblebee richness, and (c) network specialization (*H*_2_′).

Model	Variable	*χ2*	*df*	*P*
(a) Bumblebee abundance	Month	20.44	2	** < 0.0001**
	Patch isolation	6.20	1	**0.013**
	Flower density × month	9.42	2	**0.009**
(b) Bumblebee richness	Month	19.40	2	** < 0.0001**
	Patch shape complexity	2.70	1	0.100
	Flower density	6.95	1	**0.008**
(c) Network specialization (*H*_2_*′*)	Patch shape complexity	3.75	1	**0.047**
	Flower density	4.58	1	**0.032**

The *χ*^2^, the degrees of freedom (*df*) and the *P* values are calculated based on likelihood ratio tests (LRT). Variables implicated in interactions were also included in the models. Significant variables are shown in bold.

Bumblebee richness was also positively related to flower density (Table 1b; Fig. 2c) and was overall higher in July than in the other months (2.08 ± 0.68 in July vs. 1.04 ± 0.15 and 0.71 ± 0.34 in June and August, respectively; Table 1b). Patch shape complexity appeared also in the best model, although its effect was not significant (Table 1b). For bumblebee richness, we found three alternative models to the best one, with a difference in Akaike Information Criterion corrected for small sample sizes (ΔAICc) ≤ 2, but none of them included additional significant variables (SI Table S4a).

### Bumblebee community composition

**Figure 3:**
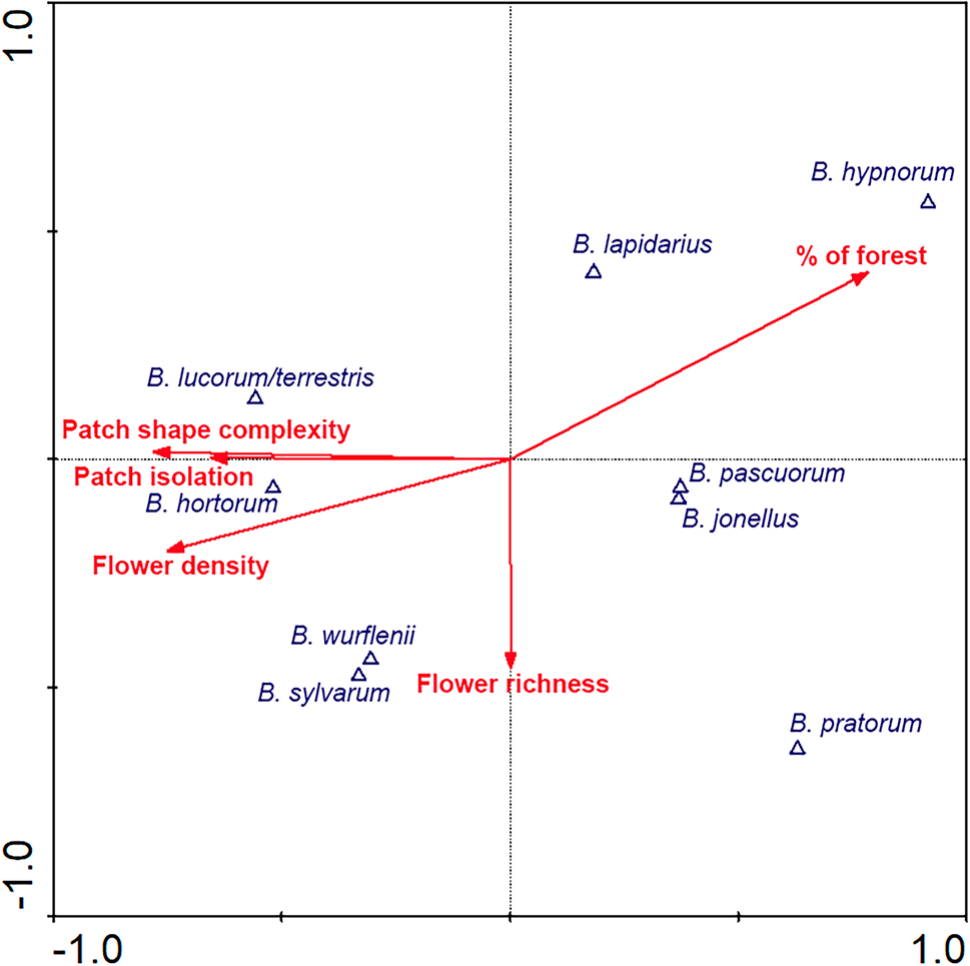
Forest fragmentation and bumblebee community composition. Canonical correspondence analysis (CCA) showing the relationships between bumblebees (blue triangles), landscape characteristics and local flowering community (red arrows). Short distances between the bumblebee species and the predictor variables in the ordination indicate high association between them. Percentage of forest in the buffer zone (*F* = 4.59, *P* = 0.001; percentage of explained variance, V%: 18%) and flower density (*F* = 2.85, *P* = 0.011; V%: 28%).

The canonical correspondence analysis (hereafter CCA) showing the relationships between bumblebee species, landscape characteristics and local flowering community was significant (first axis: *F* = 5.747, *P* = 0.004; all axes: *F* = 2.337, *P* = 0.002). The variables related to forest fragmentation and flower density varied along the first axis of ordination, whereas flower richness varied along the second axis (Fig. 3). The cumulative percentage of variance explained by the first axis was 61.5%, while the two first axes explained 84.9% of the total variance. In one direction of the first axis appeared the percentage of forest in the 500-m buffer zone, while in the other direction, the variables related to fragmented forest (patch isolation and shape complexity) appeared, together with flower density. *B. lapidarius* and specially *B. hypnorum* were positively associated with the percentage of forest in the 500-m buffer zone. The abundance of *B. lucorum/terrestris* and *B. hortorum* increased with variables related to fragmented landscape (Fig. 3), while *B. pratorum* and *B. pascuorum* increased their abundance with flower richness (Fig. 3). Forward selection indicated that the variables significantly affecting the ordination were: percentage of forest in the buffer zone (*F* = 4.59, *P* = 0.001; percentage of explained variance, V%: 18%) and flower density (*F* = 2.85, *P* = 0.011; V%: 28%).

### Network specialization

**Figure 4:**
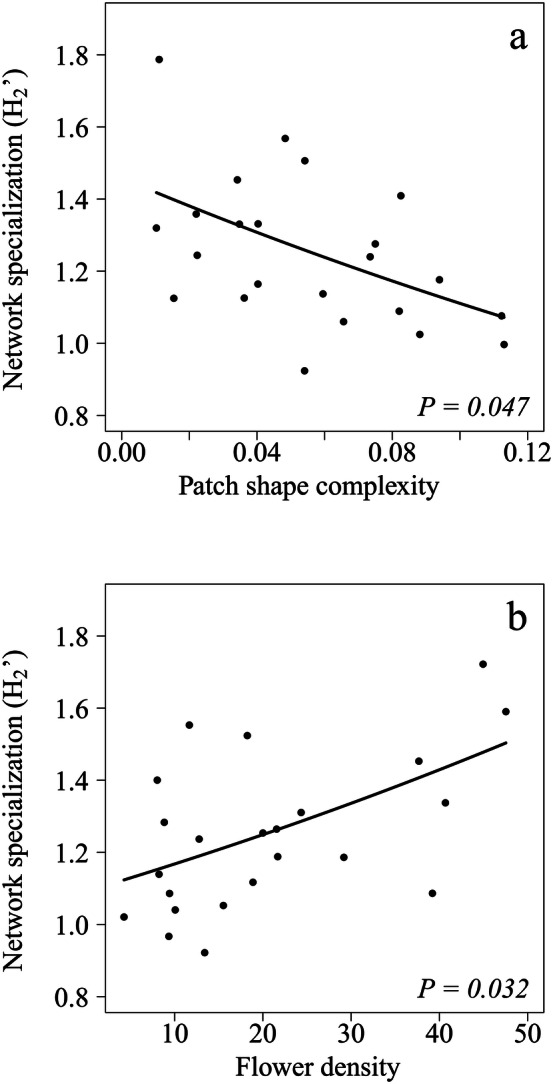
Forest fragmentation and network specialization. Partial residual plots showing the relationships between *H*_2_′ and: (**a**) patch shape complexity; and (**b**) flower density. Lines represent the estimates of the best model and the dots represent partial residuals for each study forest patch.

The best model indicated that network specialization (*H*_2_′) was negatively related to patch shape complexity (Table 1c; Fig. 4a) and positively related to flower density (Table 1c; Fig. 4b). We found four alternative models (ΔAICc ≤ 2) for this variable, but none of them included additional significant variables (SI Table S4b).

### Potential inter- and intraspecific competition

We evaluated the interactions among bumblebee species that were present in at least half of the study forest patches, i.e., *B. pascuorum, B. lucorum/terrestris, B. hypnorum* and *B. pratorum* (SI Table S3).

#### Interspecific competition

**Figure 5:**
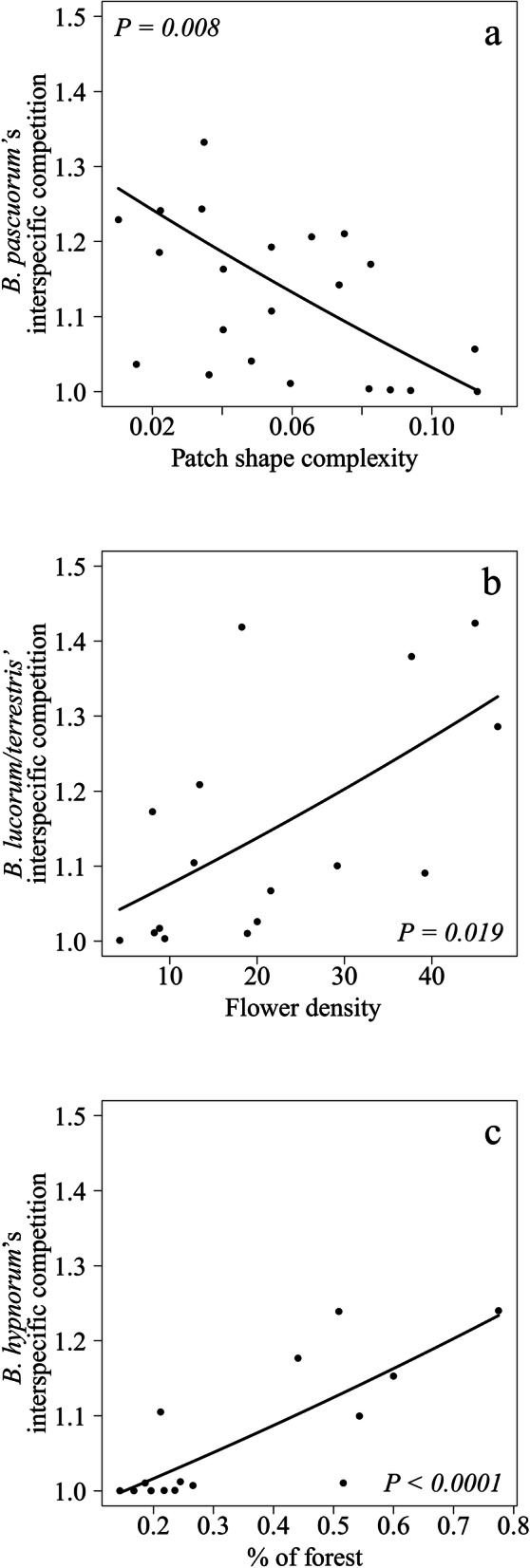
Forest fragmentation and bumblebee interspecific competition. Interspecific competition for the most common study species, (**a**) *B. pascuorum*; (**b**) *B. lucorum/terrestris* and (**c**) *B. hypnorum*. Lines represent the estimates of the best model, while dots represent partial residuals for each study forest patch.

The potential for interspecific competition significantly decreased with patch shape complexity for *B. pascuorum* (Table 2a; Fig. 5a), while it significantly increased with flower density for *B. lucorum/terrestris* (Table 2a; Fig. 5b), and with the percentage of forest in the landscape for *B. hypnorum* (Table 2a; Fig. 5c). Interspecific competition for *B. pratorum* did not significantly vary with either landscape or flowering community variables (Table 2a).

**Table 2:** Results of the best models showing the relationship between the landscape characteristics and local flowering community and (a) interspecific competition (effect of a focal species on other bumblebees via shared plants), and (b) intraspecific competition (effect of a species on its conspecifics via shared plants), for each of the four most abundant species.

Model	Species	Variable	*χ 2*	*df*	*P*
(a) Interspecific competition	*B. pascuorum*	Patch shape complexity	7.11	1	**0.007**
	*B. lucorum/terrestris*	Flower density	5.46	1	**0.019**
	*B. hypnorum*	% of forest in the 500 m-buffer zone	17.93	1	** < 0.0001**
	*B. pratorum*	Patch shape complexity	1.91	1	0.167
(b) Intraspecific competition	*B. pascuorum*	Flower density	8.03	1	**0.005**
	*B. lucorum/terrestris*	Patch shape complexity	5.35	1	**0.021**
	*B. hypnorum*	Flower richness	2.36	1	0.125
	*B. pratorum*	% of forest in the 500 m-buffer zone	2.95	1	0.086

The *χ*^2^, the degrees of freedom (*df*) and the *P* values are calculated based on likelihood ratio tests (LRT). Significant variables are shown in bold.

#### Intraspecific competition

**Figure 6:**
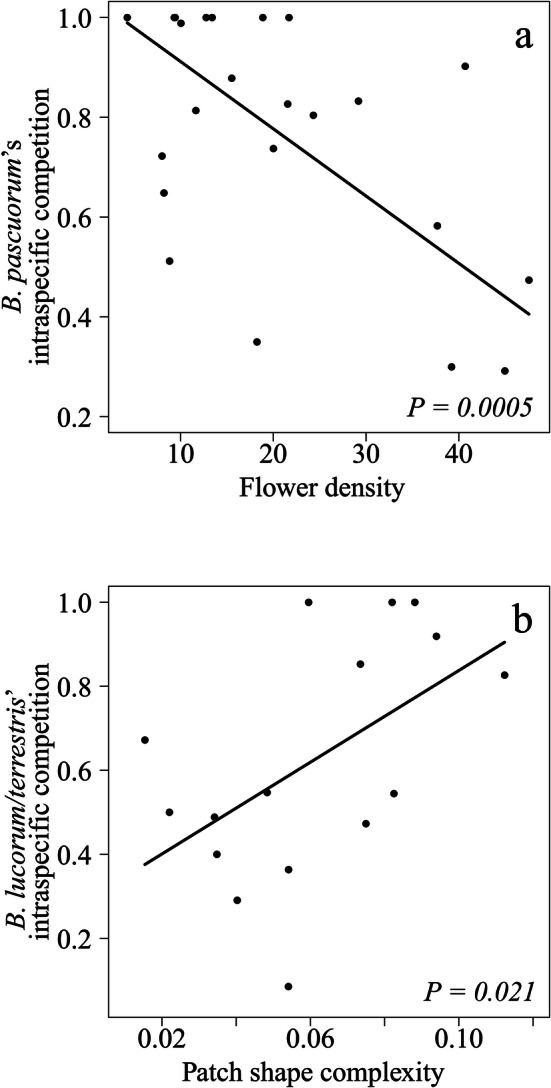
Forest fragmentation and bumblebee intraspecific competition. Intraspecific competition for the most common study species (**a**) *B. pascuorum* and (**b**) *B. lucorum/terrestris*. Lines represent the estimates of the best model, while dots represent partial residuals for each study forest patch.

Potential intraspecific competition decreased with increasing flower density for *B. pascuorum* (Table 2b; Fig. 6a), whereas it increased with patch shape complexity for *B. lucorum/terrestris* (Table 2b; Fig. 6b). For the other species, we did not find intraspecific competition to vary with either landscape or flowering community variables (Table 2b).

## Discussion

In this study, we show that forest fragmentation and the loss of flowering resources decrease bumblebee abundance and richness and drives to changes in the composition of communities, by reducing habitat-specialized species in favour of highly generalist ones. Furthermore, the increase in patch shape complexity and the reduction of flower density lead to a greater generalization of pollination networks and to changes in bumblebee’s inter- and intraspecific competitive interactions for pollination.

### Bumblebee abundance, richness and community composition along the fragmentation gradient

Overall, we recorded a total of ten bumblebee species, with a clear dominance of *B. pascuorum* and *B. lucorum/terrestris* over the other species. These ten species historically occurred in our study area or close by^43^, and most of them in similar relative abundances^44^. However, previous available data did not allow us to evaluate whether the local composition of communities has changed over time.

We found that patch isolation decreased bumblebee abundance. This negative effect of habitat isolation on flower-visiting wild pollinators is well-known and has been reported for a wide range of species in natural and semi-natural habitats^45–47^ and in crops^48,49^. It has been argued that bumblebees might be less affected by habitat isolation than other pollinators^50,51^, because their large body size allows them to fly long distances for foraging^15,46,52^. However, agreeing with our results, several previous studies on bumblebees have also shown negative effects of isolation on bumblebee abundance, richness and foraging behaviour^53,54^. The approximate maximum foraging distances of our study species are 450–500 m for *B. hypnorum*^55^, *B. lapidarius* and *B. pascuorum*^56^*,* up to 700 for *B. pratorum*^56^, and up to 800 for *B. terrestris*^56^. Interestingly, we detected significant effects of patch isolation on bumblebee abundance even with the relatively small isolation gradient of our study forest patches, with a maximum path isolation of 428 m from other forest patches (calculated as the average to the closest five patches; see methods), suggesting that increasing landscape fragmentation in this region could have very harmful effects on these important pollinators.

Besides patch isolation, flower density was positively related to bumblebee abundance and richness. Consistent with previous studies in Scandinavia^42^, the relationship of bumblebee abundance and richness with flower density varied along the flowering season, probably because bumblebee populations are much larger in the middle of the season^41,57^ and then the resources become more limiting^42^. It is also in concordance with the positive relationship between bumblebee visitation and blooming density found in other studies^15,58,59^, and with a previous study carried out in our study system that showed a strong influence of the flowering resources on the whole pollinator community^5^. It is not surprising that, in systems as the Scandinavian temperate forests, which are generally poor in flowers^60^, flower availability plays such an important role in bumblebee distribution.

As expected, not only the abundance and richness of bumblebees, but also the composition of bumblebee communities changed along the fragmentation gradient, as shown by the community composition analysis (CCA; Fig. 3). We hypothesized that species adapted to forest habitats might decrease in numbers with forest fragmentation, while species related to open areas might become more abundant^3^. Agreeing with this, we found that the abundance of *B. hypnorum* was positively related to the percentage of forest in the landscape. *B. hypnorum* is considered a species typical from northern forests^43,61^, that normally nests above ground, mainly in tree cavities^62^, and their colonies might be quite numerous in the proper habitat^55^. Conversely, the abundance of *B. lucorum/terrestris* increased with patch isolation and shape complexity. Both species, *B lucorum* and *B. terrestris*, have generalist feeding habits^39^ and prefer to nest below ground in open areas^6^. Thus, in a system as the northern temperate forest, they may thrive adequately when the landscape is fragmented and open areas increase. In addition, both species build colonies of more than 150 individuals^63,64^, with high density per square kilometer^56^, which might increase considerably their local abundance. Furthermore, the individuals of both species (*B. lucorum* and *B. terrestris)* are generally bigger than the individuals of the other species^65^. Big sizes may allow them to fly larger distances compared to other bumblebee species, as shown for *B. terrestris*^14–16,56^*,* therefore being less affected by a patchy distribution of flowering resources. On the other hand, *B. pascuorum* and *B. pratorum* responded to the increase in flower richness but also to the percentage of forest in the landscape, which might be related to their preferences for forest boundaries to nest^6,66^. *B. pascuorum* builds colonies of a hundred individuals^64^ with > 150 colonies established per square kilometer^67^, thus, it is not surprising that it is one of the most abundant species. *B. pratorum*, on the other hand, builds smaller colonies (up to 50 individuals^64^) that are less crowed in the landscape^56^, which may explain in part its lower abundance. Interestingly, the abundance of *B. hortorum*, a highly diet-specialized species with one of the longest tongues among the European bumblebees^68^, increased with flower density. This is likely because the patches with higher flower density also contain the highest abundances of flowers with long-corolla tubes, which are the flowers it visits (SI Fig. S1). Future studies might extend this work to include additional variables that are common in fragmented agricultural landscapes and that are known to affect habitat quality, as for instance pesticide use^69^.

### Trophic and competitive interactions for pollination along the fragmentation gradient

As expected, bumblebee trophic and competitive interactions were also influenced by the forest fragmentation features and the local flowering community. Particularly, we found a clear increase in network specialization with the increase in flower density, while a decrease in specialization with the increase in patch shape complexity. Likely, the positive relationship between flower density and network specialization is due to resource partitioning^19,70,71^, as increases in flower availability might increase the possibilities of bumblebee species to focus on different resources to optimize foraging and avoid competition^15^. Our results regarding patch shape complexity are in the line of other studies that also found a negative relationship between network specialization and habitat loss or disturbance^23,26^. The reasons for a decrease in network specialization as patch shape complexity increases might be twofold. First, a complex configuration of edges in the landscape might modulate habitat suitability and influence community composition, resulting in a reduction in diet specialized species in favour of more generalist ones^8,72^. Second, bumblebee species could become more generalist if landscape edges hinder their movement^53,73^ and they widen their diet to profit from local resources^39,74^. Since plant–pollinator interactions are known to vary considerably between years, affecting network structure^75,76^, future studies might evaluate the inter-annual variability of pollination networks along fragmentation gradients to understand whether forest fragmentation affects the stability of interactions through time.

Forest fragmentation and flower density also influenced bumblebees’ competitive interactions for flowering resources in a highly species-specific manner but in close relation to the changes that occurred in the community along the fragmentation gradient (CCA; Fig. 3). Same as their abundance, we found that the potential effect of *B. hypnorum* on other bumblebees through shared plants was higher in habitats with a higher proportion of forest in the surroundings, which agrees with the habitat specialization of this forest species^43,61,62^. In addition, we found that the most common species, *B. lucorum/terrestris* and *B. pascuorum* (together > 60% of total visits), were affected by the same variables (patch shape complexity and flower density) but in different directions. Potential competitive interactions in these species reflected well their patterns of distribution in the landscape. Thus, the CCA showed that while *B. lucorum/terrestris* was favored in patches with complex shapes and high flower density, the abundance of *B. pascuorum* was more related to the percentage of forest in the landscape (Fig. 3). Accordingly, as the density of flowers increased *B. lucorum/terrestris* had a stronger effect on other bumblebees via shared plants, while the intraspecific competition in *B. pascuorum* decreased as its abundance was lower in dense patches. Similarly, *B. lucorum/terrestris*’ instraspecific competition was higher in more complex patches, where the influence of *B. pascuroum* on other species was low. Therefore, overall it seems that relative abundance is determining competitive interactions in this system, where the most abundant species show the stronger effects on the less abundant ones, and intraspecific competition increases as species’ abundance increases^36^. This was to be expected, because all these four species are diet generalists^3,77^. In more specialized species, other factors also might modulate their competitive interactions, as for instance differences in tongue length that allow some species to exploit resources than others cannot^78^ or trait differences among species that may shape the strength of their competition^79^.

Regarding the winners and losers along the forest fragmentation gradient in our system, our results suggest that the potential of bumblebees to influence others via shared feeding plants might be highly linked to their capabilities to success at certain levels of forest fragmentation. As a consequence, *B. lucorum/terrestris* might be a better competitor than *B. hypnorum* and *B. pascuorum* in fragmented Scandinavian temperate forests. It might be highlighted though, that our results for intra- and interspecific competition were based on the visitation patterns to plant species, but we do not have information on whether these potential competitive interactions actually have any effect on bumblebee fitness, because measuring the performance of insect populations in the field is very difficult. The challenge of futures studies will be to understand whether these competitive relationships that arise from landscape modifications have impact on the reproductive success of the bumblebees or the plants they feed on.

## Conclusion

We found that forest fragmentation reduces overall bumblebee abundance and richness through isolation and changes in food availability, and modifies community composition by reducing forest-specialized species and enhancing highly generalist species. In addition, forest fragmentation generalizes pollination networks and influences competition among bumblebee species, with highly generalist species outcompeting habitat specialized ones in fragmented areas. Understanding these mechanisms is fundamental to evaluate the real effects of fragmentation on the pollination services provided by wild bumblebees in northern agricultural landscapes.

## Methods

### Study landscape and patches

**Figure 7:**
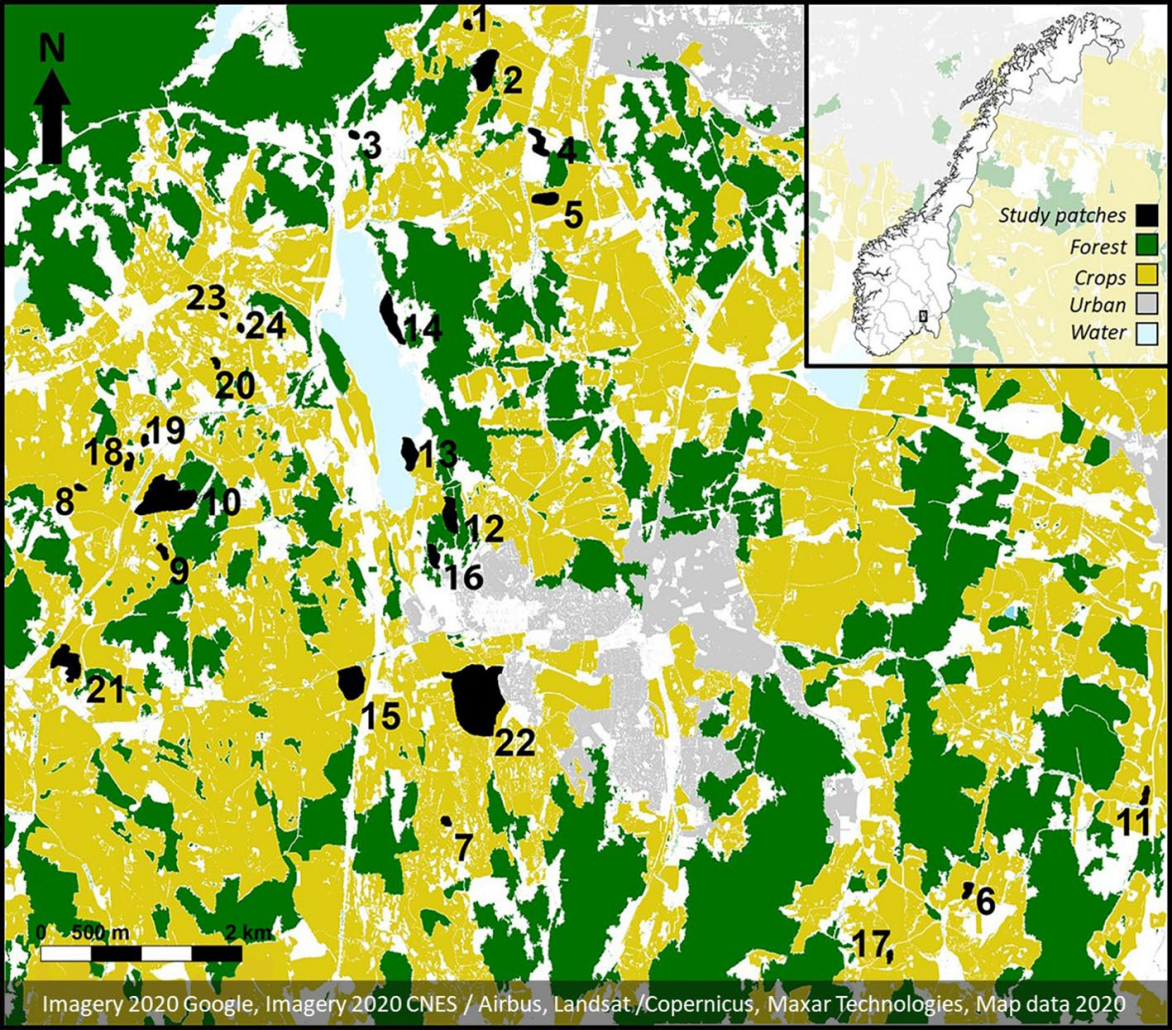
Map of the 24 study forest patches. The map shows the surroundings of the Norwegian University of Life Sciences at Ås (59°66′N, 10°79′E), about 25 km south of Oslo, Norway. Map is a modified satellite image from Google Maps (Imagery 2020 Google, Imagery 2020 CNES/Airbus, Landsat/Copernicus, Maxar Technologies, Map data 2020).

We conducted our study in the surroundings of the Norwegian University of Life Sciences at Ås (59°66′N, 10°79′E), about 25 km south of Oslo, Norway. The study area occupied 170 km^2^ and included parts of the municipalities of Frogn, Ås and Ski (Fig. 7). The landscape in the study area was dominated by cultivated farmland and crops (mainly wheat, strawberries and oilseed rape), with interspersed patches of north-temperate mixed forest. According to previous studies, the region presents a high diversity of bumblebees^43^, with *B. lucorum* and *B. pascuorum* being the most common species in the area^44^.

We used orthophotos of Norwegian mainland from *Norge i bilder* website (http://www.norgeibilder.no)^80^ to select 24 forest patches distributed across our study landscape. The study forest patches were selected to visually differ in surrounding landscape, patch size and isolation (Fig. 7), and varied in the density and richness of their local flowering communities (SI Table S1). Distance between the study forest patches varied from ca. 200 m to 11 km, with an average distance between pairs of closest study patches of 803 ± 485 m. A previous study in the area indicated that flower density increased with patch size and shape complexity and decreased as the percentage of surrounding forest increases, whereas flower richness increased with patch shape complexity^5^.

### Landscape characteristics

For each of the 24 study forest patches, we measured the following variables related to the patch or its surrounding landscape:

1. *Patch size* (ha) and *Patch perimeter* (m), estimated by using the standard toolbox in *Norge i bilder* website^80^.
2. *Patch shape complexity*, calculated as *Patch perimeter*/*Patch size*; larger edges in relation to the size indicate more complex shapes.
3. *Patch isolation*, as the mean distance of a study forest patch to the five closest forest patches (independently on whether they were study patches or not). We set isolation to zero for four patches that were connected to other forest patches through corridors (i.e., very narrow portions of forest that connect two larger portions).
4. *Percentage of cropland and forest in the 500 m-buffer zone*. We established a 500-m buffer around each sampling transect (see below for details) using ArcMap ver. 10.5^81^. We defined our buffer zone at 500 m because mean flying distance in a foraging bout for bumblebees is generally not much larger than 500 m^14,55,56,67^. We estimated the percentage of area dedicated to croplands and forest within each buffer by using the Norwegian Mapping Standard SOSI^82^.

### Field surveys

#### Bumblebee sampling

In each of the 24 study forest patches, we established a permanent bee walk transect of 100 m × 1 m to count foraging bumblebees. Transects were located within areas of the patch forest containing flowers, and as close as possible to the patch centre. Bee walks (a modified version of Goulson et al.^3^), were carried out between 09:00 and 19:00 h above 12 °C without rain or wind, from the beginning of June to the beginning of September 2007. Bee walks were conducted by five observers, each of them randomly assigned to three different study forest patches each sampling day. We visited each study forest patch 15.62 ± 2.93 different days on average, as flower availability and weather conditions prevented sampling some dates in some patches. All foraging bumblebees within a transect, as well as the plants on which they were observed, were registered as the observer walked alongside the 100 m transect. In order to observe each flower individually as walking along the transect, we spent 30–90 min at each transect per sampling day, depending on the number of available flowers which varied along the season for all the patches. On average the study forest patches were observed during 35 ± 4 min per sampling day (SI Table S5 shows averages per study forest patch). Additional information regarding sampling effort and sampling completeness is shown in SI Table S5 and SI Figure S2. Only bumblebees contacting the reproductive organs of flowers were recorded. Whenever possible, we identified bumblebee species in the field following Loken^43^. The similarities between *B. lucorum* and *B. terrestris* hindered their correct differentiation in the field^83^ and therefore, both species were included within the group *B. lucorum/terrestris*^20,84^.

#### Local flowering community

To quantify the flowering resources available for bumblebees in each study forest patch and sampling day, we established ten permanent 1 × 1 m squares within each bee walk transect at the beginning of the field season, homogeneously distributed every ten meters along the transects. The number of floral units (i.e. flowers or inflorescences depending on the species) within the squares was counted after bee walks. Plant identification followed Lid^85^. For plant species with very low abundance that appeared in the transect but not in any of the squares, all the floral units occurring along the transect were counted. For each plant species, we estimated the number of open flowers/m^2^, either by averaging the number of flowers recorded in the 10 sampling squares (for those plant species that appeared in the sampling squares) or by dividing the total number of flowers recorded in the whole transect by 100 m^2^ for those less abundant species that did not appear in the sampling squares. Thus, we estimated: (1) flower density, as the average number of total open floral units/m^2^ per sampling day and transect; and (2) flower richness, as the average number of flowering species per sampling day and transect.

### Standardization of bumblebee abundance and richness

As the study forest patches were sampled a different number of days (SI Table S5) and this could affect our estimates of bumblebee abundance and richness, we used a sample-based approach^86^ to obtain standardized and comparable measures of bumblebee visitation. For this, we first identified the study forest patch with the lowest number of sampling days and calculated bumblebee variables for that patch. Then, we subsampled the other study forest patches, by randomly selecting sampling days from the original databases, to equal the number of days in the patch with lower number of sampling days. We created 1,000 replicate subsampled bumblebee communities for each patch and used the average value of bumblebee variables as estimates for this patch. We used this sample-based approach to standardize the total bumblebee visits per patch and month (June, July and August), from which we defined (1) *Bumblebee abundance*, as the standardized number of visits to flowers per patch and month, and (2) *Bumblebee richness*, as the standardized number of different visiting species per patch and month. Besides, we also standardized the total number of visits separately for each bumblebee species per patch, to analyse community composition (see below in “Statistical analyses” section). See SI Tables S2 and S3 for registered and standardized data on total abundance and richness per month and patch, and species abundance per patch, respectively.

### Network metrics: specialization and competition

To evaluate how landscape characteristics and local flowering community influenced bumblebee–plant interactions, we built 24 quantitative interaction matrices, one for each study forest patch, with the bipartite R-package ver. 2.11^87^ in R ver. 3.5.1^88^, using the standardized number of visits of bumblebee species to plant species as link weight. To standardize link weight, we used field data on bumblebee–plant interactions for each sampling day and patch and applied a similar sample-based approach^86^ as this used to standardize bumblebee abundance and richness. In this case, however, we subsampled bumblebee–plant interaction networks instead of single values of bumblebee species or richness. Then, we used the 24 standardized interaction matrices to calculate the following indices as measures of pollination specialization and inter- and intra-specific competitive interactions.

#### Network specialization (*H*_2_′)

To compare specialization across interaction networks along the fragmentation gradient, we calculated the specialization at the network level (*H*_2_′)^89^ for each study forest patch. This index varies from 0 (no specialization) to 1 (perfect specialization) and is largely unaffected by network size^89^*.*

#### Potential intra- and interspecific competition

To calculate the potential competition between bumblebees via their shared feeding plants, we used an index defined by Müller^90^ (Müller’s index, hereafter). The Müller’s index has been used to assess the potential for apparent competition between species of the same trophic level via shared natural enemies^90,91^ and more recently, for plant species sharing pollinators^79,92^ and pollinators sharing feeding plants^93^. In our case, it quantifies the potential of one bumblebee species to influence others via their shared diet (plant species)^93^. To calculate the Müller’s index, we ran the function *PAC* within the *bipartite* R-package^87^, separately for each study forest patch. Then, for each patch, we defined two indices to be related to the landscape characteristics and the local floral community: (1) potential for *Interspecific competition*, and (2) potential for *Intraspecific competition.* Further details about the calculation of these indices can be found in Supplementary Methods.

### Statistical analyses

To evaluate how standardized bumblebee abundance and richness were related to forest fragmentation, we performed separate generalized linear mixed models (GLMM, r-package *lme4*^94^), while to study the effect of forest fragmentation on network specialization (*H*_2_*′*) and inter- and intraspecific bumblebee competition we fitted separate generalized linear models (GLM, r-package *stats* of R v.3.6^88^). We included Patch size, Patch shape complexity, Patch isolation, Percentage of forest in the 500-m buffer and Flower density and richness as predictor variables in the full models, because previous variance inflation factor (VIF) analysis excluded the other variables described in “Landscape characteristics” and “Local flowering community” sections due to collinearity (VIF values > 3)^95^. In the GLMMs, we additionally included the sampling month (June, July and August), along with its interactions with the other variables, and the identity of the study forest patch as a random factor to account for pseudoreplication. We ran the analyses of inter- and intraspecific competition separately for each of the four most abundantly distributed bumblebee species: *B. pascuorum, B. lucorum/terrestris, B. hypnorum* and *B. pratorum* (SI Table S3). We used: (1) Poisson distributions (link log) for the models of bumblebee abundance, after checking for the absence of overdispersion^95^, (2) gamma distribution (link log) for *H*_2_*′* and interspecific competition, and (3) Gaussian (link identity) for intraspecific competition, as these last models fulfilled the assumptions of normality (function *lillie.test* in r-package *nortest* v.1.0–4^96^). Both for the GLMMs and the GLMs, we conducted automatic model selection based on AICc (function *dredge*, r-package *MuMIn*^97^) to select the most parsimonious model. Based on sampled size and to avoid over-parametrization, we limited the maximum number of predictor variables to two in the case of *H*_2_*′* and to one in the case of inter- an intraspecific competition. Best models are presented in the text and if there was any alternative model (with ΔAICc ≤ 2), it is shown in the SI Table S4. Significances are based on likelihood ratio tests (LRT).

To study how landscape characteristics and local flowering community influenced bumblebee community composition, we used canonical correspondence analysis (CCA; CANOCO v.4.5^98^). We used the same predictor variables than in the other analyses, and the response variables were the standardized abundance per study forest patch of each bumblebee species registered, with the exception of *B. soroeensis*, for which we only registered one visit in the whole study period, and therefore it was considered as an incidental visit. We used 1,000 Monte Carlo permutations to assess statistical significance of the association between the identity of the bumblebees and the predictor variables. First, we obtained the significance of the whole ordination and the first axis. Second, we used forward selection to test the relationships between each predictor variable and the composition of bumblebee species.

## Supplementary information

Supplementary information
