# Supplementary material for: Forest fragmentation modifies the composition of bumblebee communities and modulates their trophic and competitive interactions for pollination
Source: Sci Rep. 2020 Jul 2;10:10872. doi: 10.1038/s41598-020-67447-y (PMC7331617; doi:10.1038/s41598-020-67447-y)
Supplement: Supplementary file 1 — Supplementary information [file 41598_2020_67447_MOESM1_ESM.docx]

**Forest fragmentation modifies the composition of bumblebee communities and modulates their trophic and competitive interactions for pollination**

**Carmelo Gómez-Martínez^1,*^, Anne Lene T.O. Aase^2^, Ørjan Totland^3^, Javier Rodríguez-Pérez^4^, Tone Birkemoe^2^, Anne Sverdrup-Thygeson^2^, and Amparo Lázaro^1^.**

^1^Mediterranean Institute for Advanced Studies (UIB-CSIC). Global Change Research Group. C/ Miquel Marquès 21, 07190, Esporles, Balearic Islands, Spain.

^2^Norwegian University of Life Sciences, Faculty of Environmental Sciences and Natural Resource Management, P.O. Box 5003, 1432 Ås, Norway.

^3^University of Bergen. Department of Biological Sciences. P.O. Box 7800, 5020 Bergen, Norway.

^4^IMAB (Institute for Multidisciplinary Research in Applied Biology) and Dpto. Ciencias del Medio Natural, Centro Jerónimo de Ayanz, Universidad Pública de Navarra (UPNA), Campus Arrosadía, 31006, Pamplona, Navarra, Spain.

**Table S1**. **Landscape characteristics and local flowering community per study forest patch.** Flower richness: average number of flowering species per sampling day; Flower density: average number of total open floral units/m^2^ per sampling day; Patch size (ha); Patch isolation: average distance (in meters) from the five nearest forest patches; Patch shape complexity: patch perimeter divided by patch size; % Crops and % Forest: percentage of area dedicated to croplands and forest within a 500 meters buffer zones surrounding the sampling transects (see methods) in each study forest patch.

| **Study forest**  **patch** | **Patch size** | **Patch isolation** | **Patch shape complexity** | **% Crops** | **% Forest** | **Flower richness** | **Flower density** |
| --- | --- | --- | --- | --- | --- | --- | --- |
| 1 | 0.49 | 52.50 | 0.059 | 0.44 | 0.48 | 5.54 | 4.26 |
| 2 | 0.52 | 263.37 | 0.054 | 0.67 | 0.24 | 10.94 | 39.21 |
| 3 | 5.61 | 0.00 | 0.022 | 0.19 | 0.51 | 10.73 | 8.23 |
| 4 | 2.27 | 94.07 | 0.040 | 0.70 | 0.28 | 9.77 | 24.33 |
| 5 | 1.53 | 145.01 | 0.035 | 0.76 | 0.23 | 14.87 | 20.01 |
| 6 | 1.03 | 222.59 | 0.106 | 0.60 | 0.37 | 9.69 | 8.21 |
| 7 | 0.59 | 177.55 | 0.048 | 0.74 | 0.24 | 8.00 | 18.24 |
| 8 | 0.38 | 173.80 | 0.088 | 0.81 | 0.17 | 10.80 | 18.87 |
| 9 | 0.39 | 133.66 | 0.073 | 0.56 | 0.43 | 16.54 | 37.69 |
| 10 | 72.98 | 43.27 | 0.010 | 0.22 | 0.77 | 17.79 | 40.68 |
| 11 | 0.56 | 428.45 | 0.082 | 0.75 | 0.22 | 13.00 | 9.43 |
| 12 | 2.20 | 0.00 | 0.054 | 0.28 | 0.60 | 12.76 | 8.02 |
| 13 | 3.56 | 159.44 | 0.036 | 0.24 | 0.37 | 7.38 | 9.34 |
| 14 | 5.39 | 0.00 | 0.022 | 0.11 | 0.52 | 13.19 | 10.06 |
| 15 | 6.30 | 86.58 | 0.015 | 0.68 | 0.21 | 7.38 | 13.41 |
| 16 | 2.54 | 0.00 | 0.034 | 0.32 | 0.54 | 10.52 | 8.82 |
| 17 | 0.26 | 382.40 | 0.112 | 0.79 | 0.20 | 17.59 | 21.57 |
| 18 | 0.70 | 107.65 | 0.075 | 0.73 | 0.27 | 17.56 | 47.55 |
| 19 | 0.30 | 178.74 | 0.082 | 0.79 | 0.19 | 12.60 | 44.96 |
| 20 | 0.40 | 41.65 | 0.066 | 0.85 | 0.14 | 17.11 | 15.53 |
| 21 | 3.21 | 93.56 | 0.040 | 0.64 | 0.34 | 18.00 | 29.18 |
| 22 | 32.15 | 264.96 | 0.011 | 0.50 | 0.44 | 7.67 | 11.66 |
| 23 | 0.11 | 133.85 | 0.113 | 0.85 | 0.14 | 18.18 | 21.69 |
| 24 | 0.24 | 96.97 | 0.093 | 0.80 | 0.19 | 17.24 | 12.78 |

**Table S2. Bumblebee abundance and richness registered per month for the 24 study forest patches**. Recorded number of bumblebee visits (bumblebee abundance) and species (bumblebee richness) for each study month (June, July, August), with the standardized values used in the analyses shown into brackets. Standardization was based on the number of sampling visits to forest patches, following the sample-based approach of Gotelli and Colwell^1^

| **Study forest** | **Sampling days** | **Bumblebee abundance** | | | |  | **Bumblebee richnes** | | |
| --- | --- | --- | --- | --- | --- | --- | --- | --- | --- |
| **patch** |  | **June** | **July** | **August** |  | | **June** | **July** | **August** |
| 1 | 13 | 4 (1) | 0 (0) | 0 (0) |  | | 2 (1) | 0 (0) | 0 (0) |
| 2 | 18 | 0 (0) | 2 (2) | 9 (0) |  | | 0 (0) | 2 (2) | 2 (0) |
| 3 | 11 | 25 (16) | 9 (6) | 9 (9) |  | | 3 (2) | 2 (2) | 3 (3) |
| 4 | 13 | 1 (0) | 4 (4) | 4 (4) |  | | 1 (0) | 1 (1) | 1 (1) |
| 5 | 15 | 8 (3) | 23 (15) | 9 (5) |  | | 2 (1) | 5 (4) | 3 (2) |
| 6 | 16 | 7 (0) | 3 (1) | 0 (0) |  | | 0 (0) | 1 (0) | 0 (0) |
| 7 | 13 | 2 (1) | 74 (50) | 5 (3) |  | | 2 (1) | 8 (6) | 1 (1) |
| 8 | 15 | 4 (2) | 5 (2) | 0 (0) |  | | 2 (1) | 2 (1) | 0 (0) |
| 9 | 13 | 1 (1) | 22 (14) | 7 (3) |  | | 1 (1) | 2 (2) | 1 (1) |
| 10 | 14 | 9 (4) | 20 (13) | 0 (0) |  | | 3 (2) | 2 (2) | 0 (0) |
| 11 | 16 | 4 (2) | 3 (1) | 1 (1) |  | | 2 (1) | 2 (1) | 1 (1) |
| 12 | 21 | 0 (0) | 42 (15) | 5 (2) |  | | 0 (0) | 6 (4) | 2 (1) |
| 13 | 13 | 5 (5) | 4 (3) | 0 (0) |  | | 3 (2) | 1 (1) | 0 (0) |
| 14 | 16 | 27 (9) | 14 (11) | 3 (1) |  | | 2 (1) | 4 (3) | 1 (0) |
| 15 | 13 | 0 (0) | 4 (2) | 3 (2) |  | | 0 (0) | 3 (2) | 2 (1) |
| 16 | 23 | 9 (4) | 15 (6) | 4 (1) |  | | 3 (2) | 4 (2) | 4 (1) |
| 17 | 17 | 3 (2) | 4 (1) | 3 (1) |  | | 1 (1) | 4 (1) | 2 (1) |
| 18 | 16 | 11 (5) | 157 (68) | 15 (9) |  | | 5 (2) | 8 (5) | 2 (2) |
| 19 | 15 | 8 (4) | 180 (75) | 0 (0) |  | | 4 (2) | 7 (5) | 0 (0) |
| 20 | 19 | 1 (0) | 13 (6) | 0 (0) |  | | 1 (0) | 5 (2) | 0 (0) |
| 21 | 16 | 15 (6) | 3 (2) | 6 (3) |  | | 6 (2) | 2 (1) | 2 (1) |
| 22 | 12 | 4 (2) | 1 (1) | 0 (0) |  | | 2 (1) | 2 (1) | 0 (0) |
| 23 | 17 | 1 (1) | 8 (4) | 1 (0) |  | | 2 (1) | 2 (1) | 1 (0) |
| 24 | 17 | 2 (1) | 10 (4) | 10 (5) |  | | 1 (1) | 3 (1) | 1 (1) |

**Table S3.** Bumblebee visits by different species in the 24 study forest patches. Recorded field data are followed by the standardized values into brackets. Standardization was conducted by a sample-based approach following Gotelli and Colwell^1^, based on the number of sampling days.

| **Study forest**  **patch** | **Sampling days** | ***Bombus hortorum*** | ***Bombus hypnorum*** | ***Bombus jonellus*** | ***Bombus lapidarius*** | ***Bombus lucourm/terrestris*** | ***Bombus pascuorum*** | ***Bombus pratorum*** | ***Bombus soroeensis*** | ***Bombus sylvarum*** | ***Bombus wurflenii*** |
| --- | --- | --- | --- | --- | --- | --- | --- | --- | --- | --- | --- |
| 1 | 13 | 0 (0) | 0 (0) | 0 (0) | 0 (0) | 1 (1) | 3 (3) | 0 (0) | 0 (0) | 0 (0) | 0 (0) |
| 2 | 18 | 0 (0) | 1 (1) | 0 (0) | 0 (0) | 1 (1) | 3 (2) | 0 (0) | 0 (0) | 0 (0) | 6 (4) |
| 3 | 11 | 0 (0) | 14 (14) | 8 (8) | 0 (0) | 1 (1) | 18 (18) | 8 (8) | 0 (0) | 0 (0) | 2 (2) |
| 4 | 13 | 0 (0) | 0 (0) | 5 (4) | 0 (0) | 0 (0) | 4 (3) | 5 (4) | 0 (0) | 0 (0) | 0 (0) |
| 5 | 15 | 2 (1) | 0 (0) | 9 (7) | 3 (2) | 2 (2) | 19 (14) | 9 (7) | 1 (1) | 0 (0) | 0 (0) |
| 6 | 16 | 0 (0) | 0 (0) | 0 (0) | 0 (0) | 0 (0) | 3 (2) | 0 (0) | 0 (0) | 0 (0) | 0 (0) |
| 7 | 13 | 9 (8) | 1 (1) | 0 (0) | 14 (12) | 40 (34) | 3 (3) | 0 (0) | 0 (0) | 2 (2) | 9 (8) |
| 8 | 15 | 0 (0) | 1 (1) | 2 (1) | 0 (0) | 3 (2) | 3 (2) | 2 (1) | 0 (0) | 0 (0) | 0 (0) |
| 9 | 13 | 0 (0) | 0 (0) | 0 (0) | 0 (0) | 22 (19) | 8 (7) | 0 (0) | 0 (0) | 0 (0) | 0 (0) |
| 10 | 14 | 0 (0) | 5 (4) | 0 (0) | 4 (3) | 0 (0) | 20 (16) | 0 (0) | 0 (0) | 0 (0) | 0 (0) |
| 11 | 16 | 0 (0) | 3 (2) | 1 (1) | 0 (0) | 2 (1) | 2 (1) | 1 (1) | 0 (0) | 0 (0) | 0 (0) |
| 12 | 21 | 1 (1) | 10 (5) | 2 (1) | 15 (8) | 8 (4) | 9 (5) | 2 (1) | 0 (0) | 0 (0) | 0 (0) |
| 13 | 13 | 4 (3) | 0 (0) | 0 (0) | 0 (0) | 0 (0) | 4 (3) | 0 (0) | 0 (0) | 0 (0) | 1 (1) |
| 14 | 16 | 0 (0) | 1 (1) | 1 (1) | 0 (0) | 0 (0) | 42 (28) | 1 (1) | 0 (0) | 0 (0) | 0 (0) |
| 15 | 13 | 0 (0) | 1 (1) | 2 (2) | 0 (0) | 2 (2) | 1 (1) | 2 (2) | 0 (0) | 0 (0) | 0 (0) |
| 16 | 23 | 0 (0) | 7 (3) | 0 (0) | 9 (4) | 1 (0) | 9 (4) | 0 (0) | 0 (0) | 1 (0) | 1 (0) |
| 17 | 17 | 0 (0) | 3 (2) | 1 (1) | 0 (0) | 3 (2) | 3 (2) | 1 (1) | 0 (0) | 0 (0) | 0 (0) |
| 18 | 16 | 16 (11) | 2 (1) | 4 (3) | 5 (3) | 56 (39) | 60 (41) | 4 (3) | 0 (0) | 6 (4) | 34 (23) |
| 19 | 15 | 20 (15) | 3 (2) | 2 (1) | 13 (10) | 99 (73) | 39 (28) | 2 (1) | 0 (0) | 1 (1) | 11 (8) |
| 20 | 19 | 4 (2) | 1 (1) | 0 (0) | 1 (1) | 0 (0) | 7 (4) | 0 (0) | 0 (0) | 0 (0) | 1 (1) |
| 21 | 16 | 0 (0) | 0 (0) | 5 (3) | 3 (2) | 2 (1) | 4 (3) | 5 (3) | 0 (0) | 1 (1) | 9 (6) |
| 22 | 12 | 0 (0) | 1 (1) | 0 (0) | 0 (0) | 0 (0) | 4 (4) | 0 (0) | 0 (0) | 0 (0) | 0 (0) |
| 23 | 17 | 0 (0) | 0 (0) | 1 (1) | 0 (0) | 0 (0) | 9 (6) | 1 (1) | 0 (0) | 0 (0) | 0 (0) |
| 24 | 17 | 0 (0) | 0 (0) | 4 (3) | 0 (0) | 12 (8) | 2 (1) | 4 (3) | 0 (0) | 0 (0) | 0 (0) |

**Table S4**. Akaike Information Criterion corrected for minimum sample sizes (AICc, Calcagno and de Mazancourt 2010) for best (italics) and the alternative models (ΔAIC < 2). a) Bumblebee richness; and b) Network specialization (*H_2_*’). *P*-values of the variables in the best and the alternative models are also shown (significant in bold)

| **Response variable** | **Best and Alternative models** | ***P*** | **AICc** |
| --- | --- | --- | --- |
| a) Bumblebee richness | *Patch shape complexity + Flower density* *+ Month* | 0.10; **0.008**; **> 0.0001** | 198.5 |
|  | Patch isolation + Flower density + Month | 0.12; **0.010**; **> 0.0001** | 198.7 |
|  | Flower density + Month | **0.02**; **> 0.0001** | 198.8 |
|  | Patch shape complexity + Patch isolation + Flower density + Month | 0.30; 0.36; **0.007**; **> 0.0001** | 200.1 |
| b) Network specialization (H_2_^’^) | *Patch shape complexity + Flower density* | 0.047; **0.032** | 5.2 |
|  | Flower density + % of forest | 0.054; 0.067 | 5.6 |
|  | Flower density | 0.12 | 6.6 |
|  | % of forest | 0.15 | 7.0 |
|  | Patch shape complexity | 0.16 | 7.2 |

**Table S5**. **Sampling effort and sampling completeness for each of the study forest patches**. Sampling days: number of days that each study forest patch was sampled; Mean sampling time (min): average sampling time per sampling day; Total sampling time (min): total time that each study forest patch was sampled during the whole study; Sampling completeness: Estimation of the percentage of the bumblebee community that was registered, calculated by means of the Chao 1 asymptotic species richness estimator^2^ (function iNEXT from r-package *iNEXT* ver. 2.0.20^3^)

| **Study forest**  **patch** | **Sampling days** | **Mean sampling time per sampling day (min)** | **Total sampling time (min)** | **Sampling completeness (%)** |
| --- | --- | --- | --- | --- |
| 1 | 13 | 32 ± 6 | 415 | 81.23 |
| 2 | 18 | 46 ± 22 | 819 | 67.92 |
| 3 | 11 | 32 ± 6 | 350 | 84.62 |
| 4 | 13 | 40 ± 16 | 519 | 81.23 |
| 5 | 15 | 38 ± 22 | 563 | 48.39 |
| 6 | 16 | 33 ± 10 | 530 | 100.00 |
| 7 | 13 | 31 ± 2 | 400 | 46.43 |
| 8 | 15 | 37 ± 15 | 552 | 48.78 |
| 9 | 13 | 35 ± 8 | 451 | 100.00 |
| 10 | 14 | 41 ± 24 | 570 | 100.00 |
| 11 | 16 | 32 ± 3 | 502 | 48.67 |
| 12 | 21 | 32 ± 7 | 680 | 62.02 |
| 13 | 13 | 31 ± 3 | 401 | 61.91 |
| 14 | 16 | 34 ± 9 | 545 | 76.18 |
| 15 | 13 | 31 ± 2 | 401 | 35.13 |
| 16 | 23 | 33 ± 6 | 770 | 67.64 |
| 17 | 17 | 35 ± 8 | 593 | 80.96 |
| 18 | 16 | 39 ± 13 | 618 | 58.72 |
| 19 | 15 | 36 ± 9 | 538 | 65.57 |
| 20 | 19 | 35 ± 14 | 663 | 39.75 |
| 21 | 16 | 41 ± 17 | 649 | 81.02 |
| 22 | 12 | 39 ± 23 | 468 | 81.37 |
| 23 | 17 | 32 ± 3 | 542 | 100.00 |
| 24 | 17 | 31 ± 4 | 531 | 58.62 |

**Figure S1** Visits by the long-tongued bumblebee *Bombus hortorum*, the total number of flowers recorded, and the number of flowers with long-corolla tubes in the 24 study forest patches. Forest patches with higher number of visits by *B. hortorum* coincide with those with higher number of long-corolla flowers, and are also the patches with overall higher total number of flowers.

**
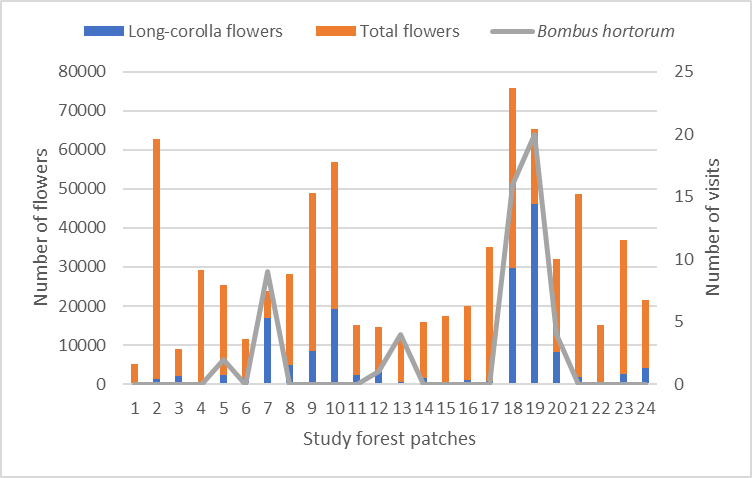
**

**Figure S2** Rarefaction curves for the number of species registered in each study forest patch. X-axis is the accumulated sampling time; Y-axis is the number of species; Solid lines indicate observed bumblebee richness; dashed lines indicated expected bumblebee richness with increasing sampling effort. Shaded area showed the bootstrapping 95% confidence interval. Value in top-left corner sampling completeness is shown (i.e., an estimation of the percentage of the bumblebee community that was registered^2^).

**
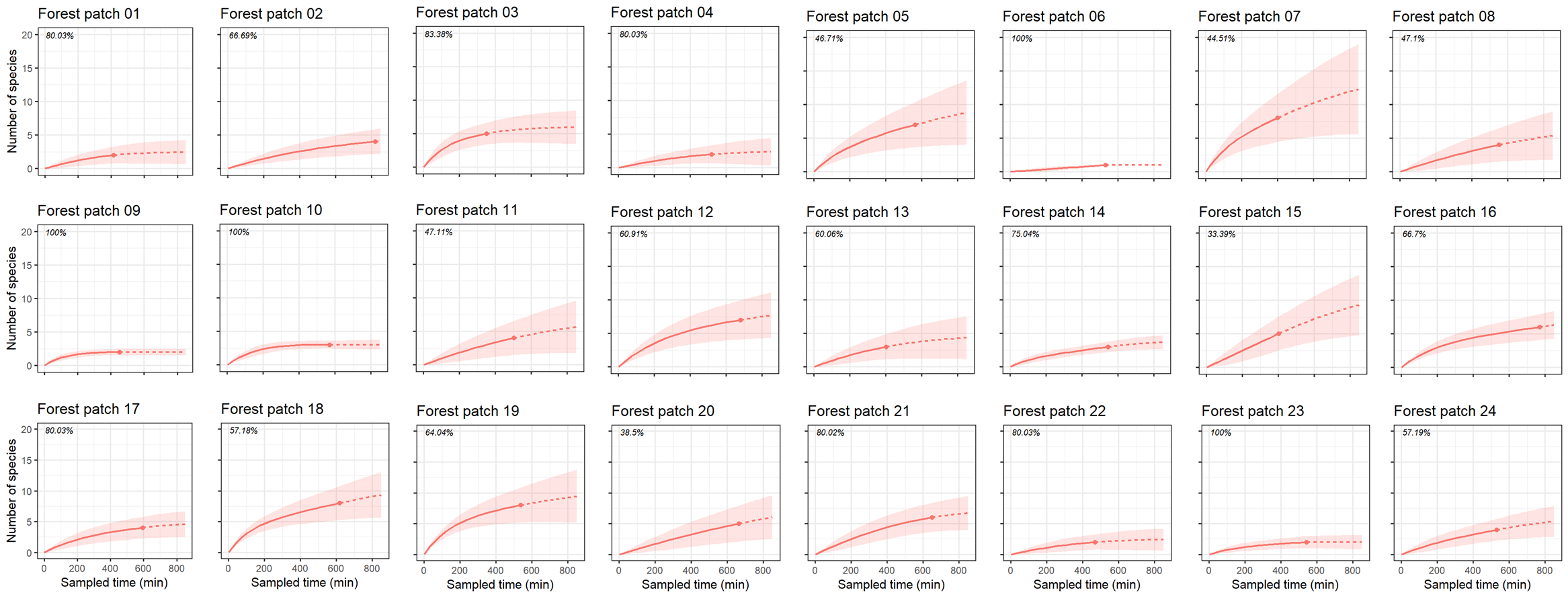
**

**SUPPLEMENTARY METHODS**

**Müller’s Index calculation**

The Müller’s index^4^ was calculated using the function *PAC* within the *bipartite* R-package^5^, where the function was defined to estimate the potential for apparent competition through a matrix with the species of the lower trophic level (the ones to quantify the potential for apparent competition) placed in rows, and the species of the higher trophic level (the ones sharing interactions with the species in the lower trophic level) placed in columns.

In this case, we used the Müller’s index to study potential inter- and intraspecific (with all bumblebee species that coexist in time and space) competition via the shared plant species they feed on. Therefore, we transposed the standardized matrices placing bumblebee species (higher trophic level) in rows and plant species (lower trophic level) in columns. In this way, Müller’s index calculates how much one bumblebee species (*acting* species) influence other bumblebee species (*target* species) via shared interactions as:

$$d_{ij}= \sum_{k} \left[ \frac{\alpha_{ik}}{\sum_{l} \alpha_{il}}x\frac{\alpha_{jk}}{\sum_{m} \alpha_{mk}} \right]$$

Where $\alpha_{ik}$ is the number of interactions of the *target* bumblebee species *i* with the plant species *k* (being *l* the total number of plants interacting with the *target* bumblebee species), and *α_jk_* represents the number of interactions of the *acting* bumblebee species *j* with the plant species *k* (*m* being the total number of bumblebees with which plant *k* interacts). This index varies between 0 (no plants shared) and 1 (all plants shared). A higher value of Müller’s index indicates a greater potential for the *acting* bumblebee species to influence the *target* bumblebee species via shared plants.

The outcome of the *PAC* function was a *k* x *k* matrix with the same bumblebee species in rows and columns. The values of each cell represent the potential influence of the species in the column on the species in the row through shared feeding plants. Diagonal values estimate the extent of intraspecific competition via shared plants they feed on^5^. We ran this function separately for each study forest patch. Then, with the 24 *PAC* matrices, we defined two indices per study forest patch, one related to interspecific competition and another related to intraspecific competition:

1) Potential for *Interspecific competition.* For each study forest patch, we averaged the values by column (not counting the ones in the diagonal) obtaining the mean effect that the focal bumblebee species (*acting* species) had on the rest of the species in the network^5-7^

2) Potential for *Intraspecific competition.* We used the values in the diagonal of the matrix to estimate the extent of intraspecific competition for each study forest patch and focal bumblebee species^5^.

To understand the effects of landscape fragmentation on inter- and intraspecific competition, these two indices were then related to the landscape characteristics and the local flowering community.

**REFERENCES**

1 Gotelli, N. J. & Colwell, R. K. Quantifying biodiversity: procedures and pitfalls in the measurement and comparison of species richness. *Ecol. Lett.* **4**, 379-391, doi:10.1046/j.1461-0248.2001.00230.x (2001).

2 Chao, A. *et al.* Rarefaction and extrapolation with Hill numbers: a framework for sampling and estimation in species diversity studies. *Ecological monographs* **84**, 45-67 (2014).

3 Hsieh, T., Ma, K. & Chao, A. iNEXT: iNterpolation and EXTrapolation for species diversity. *R package version* **2**, 1-18 (2016).

4 Muller, C. B., Adriaanse, I. C. T., Belshaw, R. & Godfray, H. C. J. The structure of an aphid-parasitoid community. *J. Anim. Ecol.* **68**, 346-370, doi:10.1046/j.1365-2656.1999.00288.x (1999).

5 Dormann, C. F., Gruber, B. & Fründ, J. J. i. Introducing the bipartite package: analysing ecological networks. **1** (2008).

6 Carvalheiro, L. G. *et al.* The potential for indirect effects between co-flowering plants via shared pollinators depends on resource abundance, accessibility and relatedness. *Ecol. Lett.* **17**, 1389-1399, doi:10.1111/ele.12342 (2014).

7 Bergamo, P. J. *et al.* The potential indirect effects among plants via shared hummingbird pollinators are structured by phenotypic similarity. *Ecology* **98**, 1849-1858, doi:10.1002/ecy.1859 (2017).
